# Supplementary material for: Cold Atmospheric Plasma Promotes the Immunoreactivity of Granulocytes In Vitro
Source: Biomolecules. 2021 Jun 17;11(6):902. doi: 10.3390/biom11060902 (PMC8235417; doi:10.3390/biom11060902)
Supplement: Supplementary file 1 [file biomolecules-11-00902-s001.zip › biomolecules-1211653-buchongcailiao/Figure S1.pdf]

Figure S1

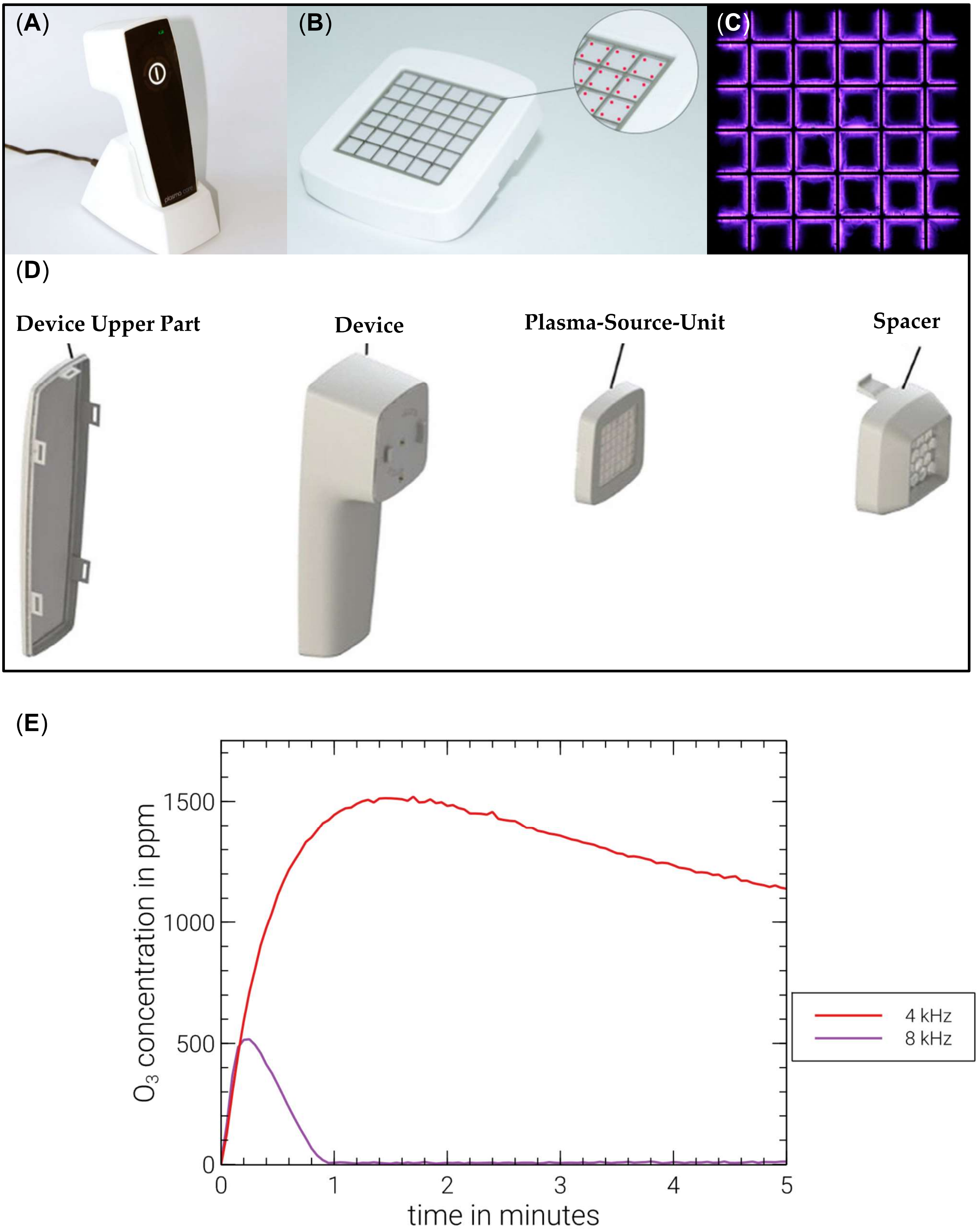

Figure S1 Design and technology of the plasma care® unit (**A**) The plasma care® unit in the charging device. (**B**) The plasma source with the electrode structured in 36 squares. (**C**) Picture of the switched-on plasma source in a darkened environment in which the characteristic purple glow of the plasma is visible. (**D**) The four essential components of the device. The upper part of the housing with touch operation, the device housing containing the electronics, the plasma source, which generates the plasma, and the sterile disposable spacer (Image courtesy of Terraplasma Medical GmbH, Garching, Germany). (**E**) Ozone (O_3) values in parts per million (ppm) produced by the plasma care® device using variable frequencies of 4 kHz and 8 kHz, 3.5 kV, and a 5 min treatment time are shown. O_3 generated by the SMD device was measured in a confined volume by ultraviolet (UV) absorption spectroscopy according to [24] and provided to us by Terraplasma GmbH. O_3 is quenched by nitric oxide and nitrogen dioxide [24], as observed in the 8 kHz treatment mode.
