# Supplementary material for: Cold Atmospheric Plasma Promotes the Immunoreactivity of Granulocytes In Vitro
Source: Biomolecules. 2021 Jun 17;11(6):902. doi: 10.3390/biom11060902 (PMC8235417; doi:10.3390/biom11060902)
Supplement: Supplementary file 1 [file biomolecules-11-00902-s001.zip › biomolecules-1211653-buchongcailiao/Figure S2.pdf]

Figure S2

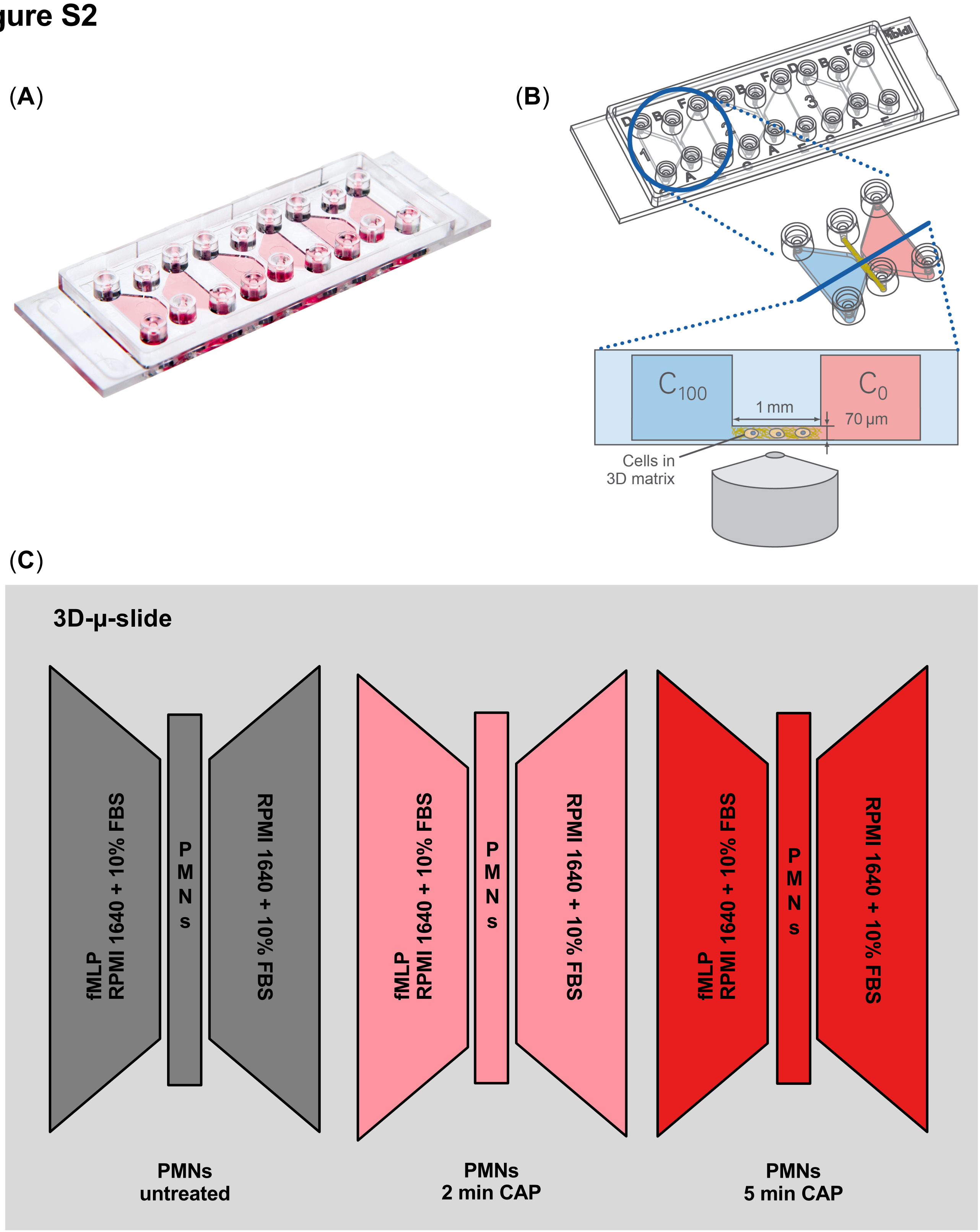

Figure S2. Structure of the ibidi© 3D-µ-slide. (**A**) Picture of the ibidi© 3D-µ-slide used for a chemotactic assay (Image courtesy of ibidi GmbH). (**B**) Schematic of the µ-slide chemotaxis principle; in this example, a 3D experiment with migratory cells in a gel matrix is shown (Image courtesy of ibidi GmbH). (**C**) Exemplary schematic of how the slide was prepared in this study.
