# Supplementary material for: Cold Atmospheric Plasma Promotes the Immunoreactivity of Granulocytes In Vitro
Source: Biomolecules. 2021 Jun 17;11(6):902. doi: 10.3390/biom11060902 (PMC8235417; doi:10.3390/biom11060902)
Supplement: Supplementary file 1 [file biomolecules-11-00902-s001.zip › biomolecules-1211653-buchongcailiao/Figure S3.pdf]

Figure S3

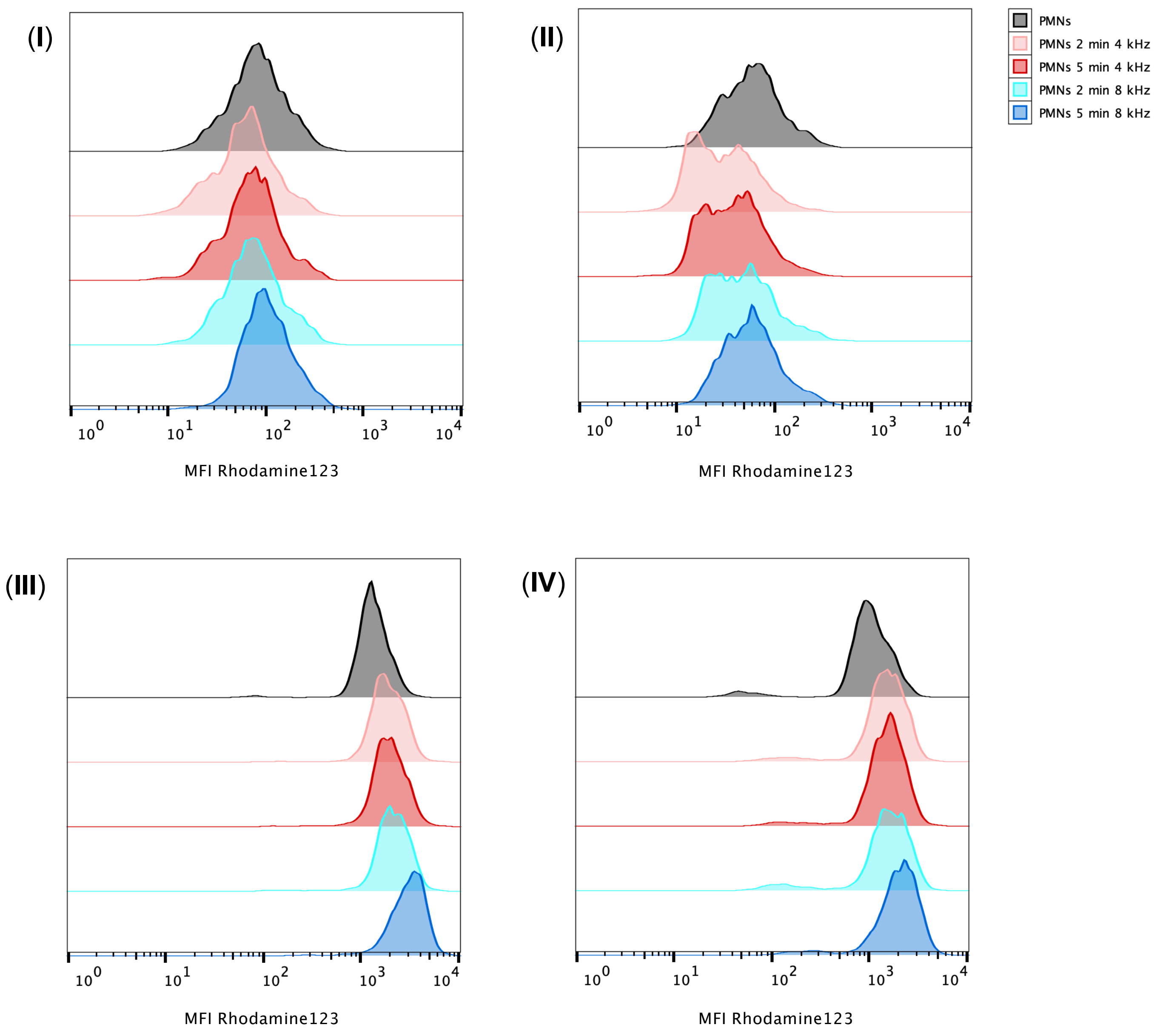

Figure S3. Representative histograms of the respiratory burst data. PMN respiratory burst activity of one exemplary donor after CAP treatment with 4 kHz or 8 kHz for 2 min and 5 min, measured by flow cytometry as median fluorescence intensity (MFI) of Rhodamine123. fMLP and TNFα stimulation (**I**) 2 h and (**II**) 6 h after CAP treatment. PMA stimulation (**III**) 2 h and (**IV**) 6 h after CAP treatment.
