# Supplementary material for: Cold Atmospheric Plasma Promotes the Immunoreactivity of Granulocytes In Vitro
Source: Biomolecules. 2021 Jun 17;11(6):902. doi: 10.3390/biom11060902 (PMC8235417; doi:10.3390/biom11060902)
Supplement: Supplementary file 1 [file biomolecules-11-00902-s001.zip › biomolecules-1211653-buchongcailiao/Table S1-S4.pdf]

Table S1. PMN migration towards fMLP or plasma-treated solution (PTS) expressed as a median (IQR).

	PMNs fMLP	PMNs 2 min PTS	PMNs 5 min PTS
**Track Length [µm] 1 – 60 min**	185.3 (167.1)	66.5 (66.9)	59.6 (41.2)
**Track Displacement X [µm] 1 – 60 min**	6.7 (54.7)	-1.5 (13.7)	-1.0 (12.9)

Table S2. Overview of microscopic results expressed as a median (IQR).

	PMNs control 4 kHz	PMNs 2 min 4 kHz	PMNs 5 min 4 kHz	PMNs control 8 kHz	PMNs 2 min 8 kHz	PMNs 5 min 8 kHz
**Track Length [µm] 1 – 30 min**	316.7 (381.6)	308.8 (359.1)	227.6 (349.6)	379.3 (369.2)	341.9 (306.9)	343.9 (341.2)
**Track Displacement X [µm] 1 – 30 min**	75.6 (171.3)	34.6 (191.2)	6.6 (158.5)	54.7 (171.9)	68.1 (167.2)	68.0 (173.0)
**Track Length [µm] 31 – 60 min**	345.4 (401.2)	350.7 (326.0)	242.5 (384.6)	328.5 (396.1)	356.9 (343.8)	335.7 (392.6)
**Track Displacement X [µm] 31 – 60 min**	46.1 (168.5)	53.1 (177.1)	2.0 (146.2)	25.4 (155.3)	63.2 (156.0)	48.3 (150.9)
**Track Length [µm] 61 – 90 min**	267.5 (353.7)	320.3 (310.5)	187.0 (281.9)	252.4 (382.7)	295.2 (334.3)	251.1 (357.4)
**Track Displacement X [µm] 61 – 90 min**	10.6 (113.1)	41.9 (156.0)	0.1 (63.8)	2.4 (98.0)	24.6 (103.6)	7.1 (106.5)
**T_{max}ROS (standardized)**	1.0 (0.3)	1.0 (0.3)	0.7 (0.5)	1.0 (0.2)	1.0 (0.1)	0.9 (0.2)
**ET_{50}NETosis (standardized)**	1.0 (0.2)	1.0 (0.2)	1.0 (0.3)	1.0 (0.1)	1.0 (0.1)	1.0 (0.1)

Table S3. ROS and RNS concentrations in plasma treated solution expressed as a median (IQR).

	RPMI	RPMI 5 min 4 kHz	RPMI 5 min 8 kHz
**MFI Rhodamine 123**	1.7 (1.0)	2.8 (3.3)	2.3 (2.5)
**H_2O_2 [µmol/L]**	0.6 (0.6)	3.4 (5.7)	3.0 (3.1)
**NO_2^- [mmol/L]**	2.1 (6.5)	35.0 (22.2)	102.9 (26.0)
**NO_3^- [mmol/L]**	73.2 (92.7)	96.9 (54.7)	145.2 (67.6)

Table S4. Overview of flow cytometric results expressed as a median (IQR).

	PMNs	PMNs<br>2 min<br>4 kHz	PMNs<br>5 min<br>4 kHz	PMNs<br>2 min<br>8 kHz	PMNs<br>5 min<br>8 kHz
**MFI Rhodamine123,<br>fMLP + TNFα,<br>2 h after CAP**	53.5<br>(40.1)	42.9<br>(31.9)	41.8<br>(40.7)	56.3<br>(27.1)	62.4<br>(39.2)
**MFI Rhodamine123,<br>fMLP + TNFα,<br>6 h after CAP**	54.2<br>(28.8)	37.9<br>(37.4)	29.0<br>(23.7)	52.1<br>(28.5)	49.7<br>(25.3)
**MFI Rhodamine123,<br>PMA,<br>2 h after CAP**	760.4<br>(773.5)	803.5<br>(883.9)	836.5<br>(800.3)	840.1<br>(1535.3)	1168.0<br>(2163.7)
**MFI Rhodamine123,<br>PMA,<br>6 h after CAP**	923.3<br>(800.6)	1129.0<br>(799.1)	1170.0<br>(920.2)	1275.0<br>(1201.7)	1672.0<br>(1895.0)
**PI^+ [%]<br>2 h after CAP**	4.9<br>(4.1)	7.0<br>(3.9)	8.6<br>(5.4)	6.8<br>(5.8)	11.4<br>(9.1)
**PI^+ [%]<br>6 h after CAP**	4.9<br>(3.5)	4.8<br>(5.9)	5.1<br>(4.3)	6.9<br>(5.9)	8.7<br>(5.9)
**MFI CD11b<br>2 h after CAP**	1164.0<br>(513.5)	1928.0<br>(790.0)	2586.0<br>(1211.0)	1535.0<br>(836.0)	2314.0<br>(898.0)
**MFI CD11b<br>6 h after CAP**	1777.0<br>(310.0)	2342.0<br>(688.0)	2756.0<br>(660.0)	2067.0<br>(343.0)	2459.0<br>(770.0)
**MFI CD62L<br>2 h after CAP**	243.6<br>(44.0)	204.9<br>(51.4)	201.7<br>(25.6)	211.6<br>(35.3)	194.9<br>(64.5)
**MFI CD62L<br>6 h after CAP**	226.0<br>(48.1)	173.7<br>(80.1)	172.6<br>(60.9)	210.0<br>(57.9)	160.9<br>(67.7)
**MFI CD66b<br>2 h after CAP**	215.9<br>(69.8)	305.9<br>(122.1)	475.2<br>(212.6)	289.6<br>(76.3)	373.1<br>(298.5)
**MFI CD66b<br>6 h after CAP**	290.2<br>(81.8)	364.8<br>(137.0)	417.1<br>(85.7)	340.2<br>(47.3)	379.6<br>(138.4)
