# Supplementary material for: Cold Atmospheric Plasma Promotes the Immunoreactivity of Granulocytes In Vitro
Source: Biomolecules. 2021 Jun 17;11(6):902. doi: 10.3390/biom11060902 (PMC8235417; doi:10.3390/biom11060902)
Supplement: Supplementary file 1 [file biomolecules-11-00902-s001.zip › Table S1-S4.pdf]

Table S1. PMN migration towards fMLP or plasma-treated solution (PTS) expressed as a median (IQR).

	PMNs fMLP	PMNs 2 min PTS	PMNs 5 min PTS
**Track Length [µm] 1 – 60 min**	185.3 (167.1)	66.5 (66.9)	59.6 (41.2)
**Track Displacement X [µm] 1 – 60 min**	6.7 (54.7)	-1.5 (13.7)	-1.0 (12.9)

Table S2. Overview of microscopic results expressed as a median (IQR).

	PMNs control 4 kHz	PMNs 2 min 4 kHz	PMNs 5 min 4 kHz	PMNs control 8 kHz	PMNs 2 min 8 kHz	PMNs 5 min 8 kHz
**Track Length [µm] 1 – 30 min**	316.7 (381.6)	308.8 (359.1)	227.6 (349.6)	379.3 (369.2)	341.9 (306.9)	343.9 (341.2)
**Track Displacement X [µm] 1 – 30 min**	75.6 (171.3)	34.6 (191.2)	6.6 (158.5)	54.7 (171.9)	68.1 (167.2)	68.0 (173.0)
**Track Length [µm] 31 – 60 min**	345.4 (401.2)	350.7 (326.0)	242.5 (384.6)	328.5 (396.1)	356.9 (343.8)	335.7 (392.6)
**Track Displacement X [µm] 31 – 60 min**	46.1 (168.5)	53.1 (177.1)	2.0 (146.2)	25.4 (155.3)	63.2 (156.0)	48.3 (150.9)
**Track Length [µm] 61 – 90 min**	267.5 (353.7)	320.3 (310.5)	187.0 (281.9)	252.4 (382.7)	295.2 (334.3)	251.1 (357.4)
**Track Displacement X [µm] 61 – 90 min**	10.6 (113.1)	41.9 (156.0)	0.1 (63.8)	2.4 (98.0)	24.6 (103.6)	7.1 (106.5)
**T_{max}ROS (standardized)**	1.0 (0.3)	1.0 (0.3)	0.7 (0.5)	1.0 (0.2)	1.0 (0.1)	0.9 (0.2)
**ET_{50}NETosis (standardized)**	1.0 (0.2)	1.0 (0.2)	1.0 (0.3)	1.0 (0.1)	1.0 (0.1)	1.0 (0.1)

Table S3. ROS and RNS concentrations in plasma treated solution expressed as a median (IQR).

	RPMI	RPMI 5 min 4 kHz	RPMI 5 min 8 kHz
**MFI Rhodamine 123**	1.7 (1.0)	2.8 (3.3)	2.3 (2.5)
**H_2O_2 [µmol/L]**	0.6 (0.6)	3.4 (5.7)	3.0 (3.1)
**NO_2^- [mmol/L]**	2.1 (6.5)	35.0 (22.2)	102.9 (26.0)
**NO_3^- [mmol/L]**	73.2 (92.7)	96.9 (54.7)	145.2 (67.6)

Table S4. Overview of flow cytometric results expressed as a median (IQR).

	**PMNs**	**PMNs 2 min 4 kHz**	**PMNs 5 min 4 kHz**	**PMNs 2 min 8 kHz**	**PMNs 5 min 8 kHz**
**MFI Rhodamine123, fMLP + TNFα, 2 h after CAP**	53.5 (40.1)	42.9 (31.9)	41.8 (40.7)	56.3 (27.1)	62.4 (39.2)
**MFI Rhodamine123, fMLP + TNFα, 6 h after CAP**	54.2 (28.8)	37.9 (37.4)	29.0 (23.7)	52.1 (28.5)	49.7 (25.3)
**MFI Rhodamine123, PMA, 2 h after CAP**	760.4 (773.5)	803.5 (883.9)	836.5 (800.3)	840.1 (1535.3)	1168.0 (2163.7)
**MFI Rhodamine123, PMA, 6 h after CAP**	923.3 (800.6)	1129.0 (799.1)	1170.0 (920.2)	1275.0 (1201.7)	1672.0 (1895.0)
**PI^+ [%] 2 h after CAP**	4.9 (4.1)	7.0 (3.9)	8.6 (5.4)	6.8 (5.8)	11.4 (9.1)
**PI^+ [%] 6 h after CAP**	4.9 (3.5)	4.8 (5.9)	5.1 (4.3)	6.9 (5.9)	8.7 (5.9)
**MFI CD11b 2 h after CAP**	1164.0 (513.5)	1928.0 (790.0)	2586.0 (1211.0)	1535.0 (836.0)	2314.0 (898.0)
**MFI CD11b 6 h after CAP**	1777.0 (310.0)	2342.0 (688.0)	2756.0 (660.0)	2067.0 (343.0)	2459.0 (770.0)
**MFI CD62L 2 h after CAP**	243.6 (44.0)	204.9 (51.4)	201.7 (25.6)	211.6 (35.3)	194.9 (64.5)
**MFI CD62L 6 h after CAP**	226.0 (48.1)	173.7 (80.1)	172.6 (60.9)	210.0 (57.9)	160.9 (67.7)
**MFI CD66b 2 h after CAP**	215.9 (69.8)	305.9 (122.1)	475.2 (212.6)	289.6 (76.3)	373.1 (298.5)
**MFI CD66b 6 h after CAP**	290.2 (81.8)	364.8 (137.0)	417.1 (85.7)	340.2 (47.3)	379.6 (138.4)
